# Impact of Grape Products on Lipid Profile: A Meta-Analysis of Randomized Controlled Studies

**DOI:** 10.3390/jcm9020313

**Published:** 2020-01-22

**Authors:** Roberta Lupoli, Paola Ciciola, Giuseppina Costabile, Rosalba Giacco, Matteo Nicola Dario Di Minno, Brunella Capaldo

**Affiliations:** 1 Department of Molecular Medicine and Medical Biotechnology, Federico II University, 80131 Naples, Italy; 2 Department of Clinical Medicine and Surgery, Federico II University, 80131 Naples, Italy; paola.ciciola@gmail.com (P.C.); giukost@libero.it (G.C.); bcapaldo@unina.it (B.C.); 3 Institute of Food Science, National Research Council, 83100 Avellino, Italy; rgiacco@isa.cnr.it; 4 Department of Translational Medical Sciences, Federico II University, 80131 Naples, Italy; dario.diminno@hotmail.it

**Keywords:** grape, polyphenol, lipid profile, meta-analysis

## Abstract

**Background**: Growing evidence shows that grape polyphenols can improve cardiovascular risk factors. Although there are clear data supporting a beneficial effect of grape supplementation on blood pressure and glucose metabolism, the effects of grape polyphenols on lipid metabolism are still controversial. **Objective**: We performed a meta-analysis of randomized controlled trials (RCTs) to assess the effect of grape products on lipid profile. **Design**: A systematic search was performed in the PubMed, Web of Science, Scopus, and EMBASE databases without any language or publication year restriction. The reference lists of all retrieved articles were manually reviewed. RCTs evaluating the impact of grape products/juice/extracts on lipid profile were included. Difference in total cholesterol (TC), high-density lipoprotein cholesterol (HDL-C), low-density lipoprotein cholesterol (LDL-C), triglycerides (TG), oxidized low-density lipoprotein cholesterol (oxLDL-C), apolipoprotein (apo) A, apo B before and after administration of grape products or placebo were expressed as mean differences (MD) with pertinent 95% confidence intervals (95% CI). The impact of clinical and demographic features on effect size was assessed by meta-regression. **Results**: The administration of grape products is associated with a significant improvement of lipid profile, as evidenced by changes in TC (MD: −7.6 mg/dL (−0.2 mmol/L); 95% CI: −10.8, −4.4; *p* < 0.001), HDL-C (MD: 1.4 mg/dL (0.04 mmol/L); 95% CI: 0.8, 1.9; *p* < 0.001, I^2^ = 74.7%, *p* < 0.001), LDL-C (−6.3 mg/dL (−0.16 mmol/L); 95% CI: −9.5, −3.0; *p* < 0.001), oxLDL-C (MD: −4.5 U/L; 95% CI: −7.5, −1.5; *p* = 0.003, I^2^ = 90.6%, *p* < 0.001), apo B (MD: −2.4 mg/dL (−0.05 µmol/L); 95% CI: −4.5, −0.3; *p* = 0.026), and TG (MD: −14.5 mg/dL (−0.16 mmol/L); 95% CI: −17.7, −11.2; *p* < 0.001) levels in subjects receiving grape products compared to placebo. With regard to the extent of the lipid-lowering effect, compared to baseline values, the highest reduction was reported for LDL-C (MD: −5.6 mg/dL (−0.14 mmol/L); 95% CI: −9.5, −1.7; *p* = 0.005) and for oxLDL-C (MD: −5.0 U/L; 95% CI: −8.8, −1.2; *p* = 0.010, I^2^ = 0%, *p* = 0.470). **Conclusions**: Grape polyphenols exert a favorable effect on lipid profile in humans by significantly reducing plasma levels of LDL-C and oxLDL-C.

## 1. Introduction

Polyphenols are a heterogeneous class of bioactive compounds mainly found in plant-based foods, such as fruits, vegetables, wholegrain cereals, and beverages, such as coffee, tea, and red wine. According to their chemical structure, five main classes of polyphenols have been identified: flavonoids, phenolic acids, lignans, stilbenes, and other minor polyphenols [1].

There is much evidence that polyphenol-rich food consumption is associated with several health benefits, namely reduction of the risk of type 2 diabetes, obesity, and cardiovascular (CV) diseases [2,3,4,5]. Of note, some classes of plant-derived polyphenols have obtained specific health claims by the European Food Safety Authority (EFSA), such as the hydroxytyrosol and its derivatives contained in extra virgin olive oil for their proven anti-oxidant action [6], and the cocoa flavanols for their favorable effect on endothelium-dependent vasodilation [7]. Over the last years, growing attention has been paid to polyphenols from grape for their potential to reduce CV risk [8,9]. Indeed, grape and its fractions (skin, flesh/pulp, seeds) contain flavonoids, resveratrol, and phenolic acids [10] to a variable extent, depending on grape’s species and geographic origin [11,12].

The cardioprotective effects of grape polyphenols are likely mediated by their ability to improve CV risk factors [13,14,15], to reduce oxidative stress [16,17] and inflammatory status [18,19], to increase the anti-oxidant capacity [18,20], and to inhibit platelet function [21,22]. In healthy subjects, grape consumption has been shown to improve flow-mediated dilation and to blunt the endothelial dysfunction caused by a high-fat diet [23]. In individuals with components of the metabolic syndrome, randomized placebo-controlled trials have shown that grape supplementation for a variable time (2–24 weeks)—provided in the form of powder, juice, or seed extracts—improved several metabolic abnormalities, significantly lowered blood pressure [24,25,26], and reduced oxidative stress markers, i.e., oxidized LDL [27]. With regard to glucose metabolism, a recent meta-analysis by Zhu et al. evaluating nine clinical trials performed in patients with type 2 diabetes mellitus (DM2) demonstrated the efficacy of resveratrol in reducing fasting glucose and improving insulin sensitivity with no relevant effect on glycated hemoglobin [28]. Similar benefits on glucose metabolism were observed after consumption of different grape products, such as seed extracts [29], juice [30], and pomace [31,32,33]. In contrast, the effects of grape polyphenols on lipid metabolism are less clear [34,35], with some studies demonstrating a significant lipid-lowering effect [20,36,37] and others failing to find any change in lipid profile [38,39,40]. Therefore, we conducted a meta-analysis of intervention cohort studies to assess the effect of grape products on lipid profile, i.e., total cholesterol (TC), high-density lipoprotein cholesterol (HDL-C), low-density lipoprotein cholesterol (LDL-C), triglycerides (TG), and main apolipoprotein (Apo) concentrations taking into account some possible confounders.

## 2. Materials and Methods

A protocol for this review was prospectively developed, reporting the specific objectives, the criteria for study selection, the outcomes, and the statistical methods.

### 2.1. Search Strategy

To identify all available studies, a systematic research of the literature published until June 2019 pertaining to the effect of grape products on lipid fractions was performed according to PRISMA (Preferred Reporting Items for Systematic reviews and Meta-Analyses) guidelines. The research was carried out in the electronic databases (PubMed, Web of Science, Scopus, and EMBASE), using the following search terms in all possible combinations: grape, lipid, cholesterol, total cholesterol, HDL cholesterol, LDL cholesterol, triglycerides, oxidized-LDL cholesterol, apolipoprotein A, and apolipoprotein B.

The search strategy was developed without any language or publication year restriction. In addition, the references of all retrieved articles were manually reviewed to find other relevant articles. In the case of missing data, study authors were contacted by e-mail to try to retrieve original data. Two authors (P.C. and G.C.) independently analyzed each article and performed the data extraction independently. In case of disagreement, a third investigator was consulted (R.L.). Discrepancies were resolved by consensus. Selection results showed a high inter-reader agreement (κ = 0.98) and have been reported according to the PRISMA flowchart (Figure S1).

### 2.2. Data Extraction and Quality Assessment

According to the pre-specified protocol, RCT studies evaluating the impact of supplementation with grape juice/extracts/products on lipid profile were included. Case-reports, case-series without a control group, reviews, and animal studies were excluded. We included in the analysis RCT studies which provide values (mean and standard deviation/standard error) of at least one variable among TC, HDL cholesterol, LDL cholesterol, triglycerides, oxidized-LDL cholesterol (oxLDL-C), apolipoprotein A (apo A), and apolipoprotein B (apo B) before and after administration of grape juice/extracts/products or placebo. In each study, data regarding sample size, major clinical and demographic characteristics of the study population, variables of interest in the intervention and placebo groups were extracted before and after grape supplement administration.

### 2.3. Statistical Analysis and Risk of Bias Assessment

Statistical analysis was carried out using Review Manager (Version 5.2, The Cochrane Collaboration, Copenhagen, Denmark) provided by the Cochrane Collaboration. Differences in changes in lipid parameters between intervention and placebo groups as well as lipid changes before and after grape supplementation were expressed as mean difference (MD) with pertinent 95% confidence intervals (95% CI) for continuous variables. TC, HDL-C, LDL-C, TG were expressed in milligrams/deciliter (mg/dL), apo A and apo B in grams/liter (g/L), oxLDL-C in U/L.

The overall effect was tested using Z scores and significance was set at *p* < 0.05. Statistical heterogeneity between studies was assessed with chi square Cochran’s Q test and with I^2^ statistic, which measure the inconsistency across study results and describe the proportion of total variation in study estimates, that is due to heterogeneity rather than sampling error. In detail, I^2^ values of 0% indicates no heterogeneity, 25% low, 25–50% moderate, and 50% high heterogeneity [41]. Publication bias was assessed by the Egger’s test and represented graphically by funnel plots of the standard difference in means versus the standard error. Visual inspection of funnel plot asymmetry was performed to address for possible small-study effect, and Egger’s test was used to assess publication bias, over and above any subjective evaluation. A *p* < 0.10 was considered statistically significant [42]. In the case of a significant publication bias, the Duval and Tweedie’s trim and fill method with the random-effect model was used to allow for the estimation of an adjusted effect size [43]. In order to be as conservative as possible, the random-effect method was used for all analyses to take into account the variability among included studies.

### 2.4. Subgroup Analyses

Differences in changes in lipid parameters in the grape supplementation group were stratified according to (*a*) the type of grape product; (*b*) the polyphenol content of supplementation; (*c*) duration of polyphenol supplementation.

### 2.5. Meta Regression Analyses

To assess whether differences among included studies could be affected by demographic variables (mean age, male gender), coexistence of traditional CV risk factors (diabetes mellitus, obesity, hyperlipidemia), and length of follow-up, we performed meta-regression analyses after implementing a regression model with difference in TC, HDL-C, LDL-C, and TG as dependent variables (*y*) and the above-mentioned co-variates as independent variables (*x*). No meta-regression analysis was performed with apo A, apo B, and oxLDL-C because of the limited number of studies reporting clinical and demographic data. This analysis was performed with Comprehensive Meta-analysis (Version 2, Biostat, Englewood, NJ, USA (2005)).

## 3. Results

After excluding duplicates, the search retrieved 699 articles. Of these studies, 655 were excluded because after scanning the title and/or the abstract they were off the topic or were reviews/comments/case reports or lacked data of interest. Twenty studies were excluded after full-length paper evaluation. One study [44] provided separate data on pre- and postmenopausal women and was considered as two separate datasets. Three studies [27,45,46] reported separate data on two intervention groups receiving different amount of grape product compared with a single placebo group. In order to avoid duplicating controls, we considered data on patients receiving the highest amount of grape product.

Thus, 24 articles (25 datasets, 618 subjects assigned to grape juice/extract/product administration and 587 subjects assigned to placebo) were included in the final analysis [27,29,32,33,34,35,44,45,46,47,48,49,50,51,52,53,54,55,56,57,58,59,60,61]. In detail, 21 studies (22 data-sets) reported data on TC, 22 studies (23 datasets) on HDL-C, 20 studies (21 datasets) on TG, 20 studies (21 datasets) on LDL-C, 4 studies (4 datasets) on oxLDL-C, 2 studies (2 datasets) on apo A, 3 studies (3 datasets) on apo B.

### 3.1. Study Characteristics

All included studies were RCTs; the major characteristics of study populations are shown in Table 1. The number of enrolled subjects varied from 8 to 50, the mean age from 20.7 to 62.0 years, and the prevalence of male gender from 0% to 100%.

Eleven studies evaluated the effect of whole grape products (grape powder or pomace) [32,33,34,44,45,50,51,53,55,59,61], five studies focused on grape juice [48,49,52,56,57], seven studies on grape seed extracts [27,29,35,46,47,54,58], and one study on the effect of grape skin extracts [60].

The presence of dyslipidemia was reported in 0–100% of patients, diabetes mellitus in 0–100% of patients, hypertension in 0–86%, smoking habit in 0–100% and previous coronary artery disease (CAD) in 0–24%. Mean body mass index (BMI) varied from 21.4 to 36.8 kg/m^2^ (mean 28.4 kg/m^2^) and waist circumference from 84.1 to 109 cm. Mean fasting glucose levels ranged from 80 to 103 mg/dL, systolic blood pressure from 113 to 134 mmHg and diastolic blood pressure from 70 to 94 mmHg.

### 3.2. Total Cholesterol, HDL Cholesterol, Triglycerides

As reported in Figure S2, we found a greater reduction in TC levels after administration of grape products as compared with placebo (MD: −7.6 mg/dl (−0.2 mmol/L); 95% CI: −10.8, −4.4; *p* < 0.001). Heterogeneity among studies was significant (I^2^ = 94.3%, *p* < 0.001) and no reduction in the overall heterogeneity was found after excluding one study at a time. In the intervention group, we found a trend toward a significant decrease in TC levels after supplementation with grape products as compared with pre-intervention TC levels (MD: −5.0 mg/dL (−0.13 mmol/L); 95% CI: −10.2, 0.1; *p* = 0.057, I^2^ = 41.9%, *p* = 0.021).

Although administration of grape products resulted in a more significant increase in HDL-C levels as compared with placebo (MD: 1.4 mg/dL (0.04 mmol/L); 95% CI: 0.8, 1.9; *p* < 0.001, I^2^ = 74.7%, *p* < 0.001, Figure S3), no significant change in HDL-C was found in the intervention group between pre- and post-supplementation levels (MD: 0.9 mg/dL (0.02 mmol/L); 95% CI: −0.3, 2.1; *p* = 0.122, I^2^ = 0%, *p* = 1.000).

We observed a greater decrease in TG levels after supplementation with grape products as compared with placebo (MD: −14.5 mg/dL (−0.16 mmol/L); 95% CI: −17.7, −11.2; *p* < 0.001, Figure S4). Heterogeneity among these studies was significant (I^2^ = 94.1%, *p* < 0.001) and no reduction in the overall heterogeneity was found after excluding one study at a time. However, we found no significant changes in TG levels between pre- and post-supplementation in the intervention group (MD: 0.60 mg/dL (0.01 mmol/L); 95% CI: −2.0, 3.2; *p* = 0.654, I^2^ = 0%, *p* = 0.881).

Meta-regression models (Table S1) showed that an increasing age was associated with a less significant improvement in HDL-C after grape product supplementation as compared with placebo (Z-value: −2.58, *p* = 0.001). None of the other clinical and demographic variables influenced differences in changes in TC, HDL-C, and TG.

### 3.3. LDL Cholesterol

**Figure 1:**
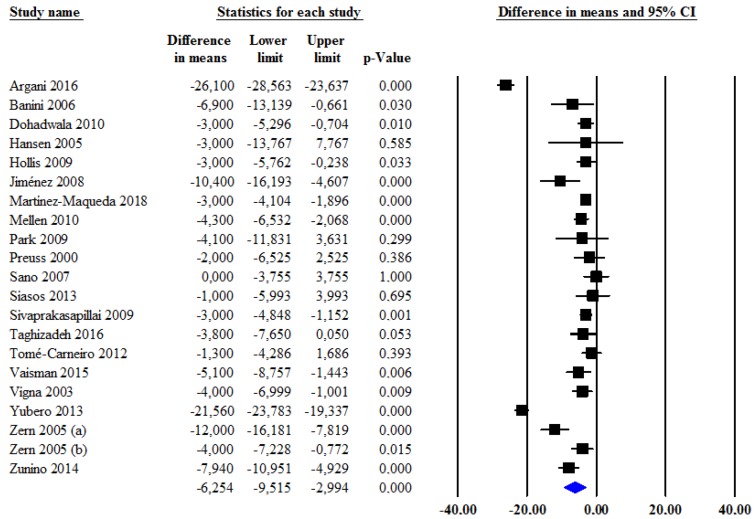
Changes in low-density lipoprotein cholesterol (LDL-C) levels after administration of grape products as compared with controls.

A significantly greater reduction in LDL-C levels was observed after administration of grape products as compared with those after the administration of placebo (MD: −6.3 mg/dL (−0.16 mmol/L); 95% CI: −9.5, −3.0; *p* < 0.001) (Figure 1). Heterogeneity among studies was significant (I^2^ = 96.3%, *p* < 0.001) and no reduction in the overall heterogeneity was found after excluding one study at a time.

Meta-regression models (Supplemental Table S1) showed that none of the evaluated clinical and demographic variables influenced differences in changes in LDL-C after grape products supplementation as compared with placebo.

**Figure 2:**
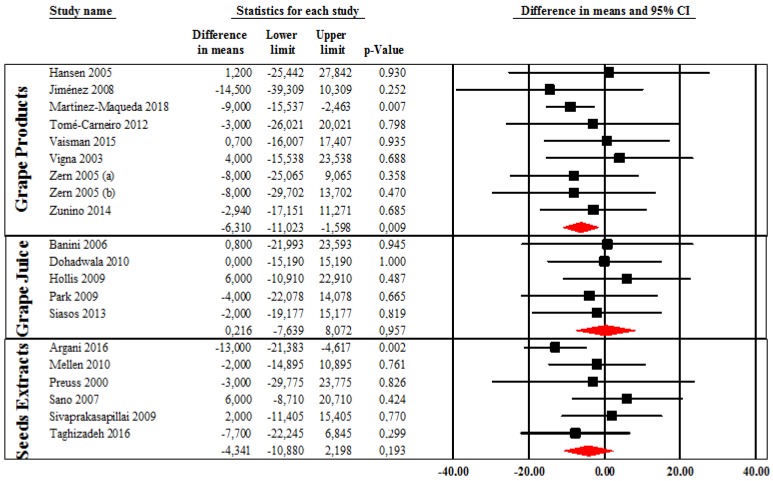
Changes in LDL-C levels in subjects taking whole grape products, grape juice, and grape seeds extracts.

In the intervention group, we found a significant decrease in LDL-C after supplementation with grape products as compared with pre-intervention LDL-C levels (MD: −5.6 mg/dL (−0.14 mmol/L); 95% CI: −9.5, −1.7; *p* = 0.005) with a non-significant heterogeneity (I^2^ = 29.1%, *p* = 0.105). Stratifying the population according to grape product types, we found a significant reduction in LDL-C levels in subjects taking whole grape products (MD: −6.3 mg/dL (−0.16 mmol/L); 95% CI: −11.0, −1.6; *p* = 0.009, I^2^ = 0%, *p* = 0.901) while no significant difference in LDL-C was found after supplementation with grape seeds extracts (MD: −4.3 mg/dL (−0.11 mmol/L); 95% CI: −10.9, 2.2; *p* = 0.193, I^2^ = 28.9%, *p* = 0.218) and with grape juice (MD: 0.21 mg/dL (0.01 mmol/L); 95% CI: −7.6, 8.1; *p* = 0.957, I^2^ = 0%, *p* = 0.948) (Figure 2). Only one study [60] reported a significant reduction in LDL-C after consumption of grape skin extracts. Moreover, analyzing data according to polyphenol content of the supplements used in each study, we found a significant reduction in LDL-C levels in subject taking supplements with a polyphenol content >400 mg/day (MD: −5.8 mg/dL (−0.15 mmol/L); 95% CI: −10.7, −0.8; *p* = 0.022, I^2^ = 0%, *p* = 0.662), but not in those receiving supplements with a lower polyphenol content (MD: −4.3 mg/dL (−0.11 mmol/L); 95% CI: −11.9, 3.2; *p* = 0.260, I^2^ = 57.2%, *p* = 0.013). Both subjects receiving polyphenol supplementation for <8 weeks and those treated for ≥8 weeks showed a significant reduction in LDL-C levels compared to control subjects (−4.7 mg/dL (−0.12 mmol/L); 95% CI: −6.3, −3.1; *p* < 0.001 and −7.4 mg/dL (−0.19 mmol/L); 95%CI: −13.7, −1.1; *p* = 0.02).

A significantly greater reduction in oxLDL-C levels was observed after administration of grape products as compared with placebo (MD: −4.5 U/L; 95% CI: −7.5, −1.5; *p* = 0.003, I^2^ = 90.6%, *p* < 0.001), as well as after supplementation as compared to baseline values (MD: −5.0 U/L; 95% CI: −8.8, −1.2; *p* = 0.010, I^2^ = 0%, *p* = 0.470).

### 3.4. Apo A and Apo B

We found no significant change in apo A levels both considering administration of grape products vs. placebo (MD: 7.7 mg/dL (2.8 µmol/L); 95% CI: −7.5, 22.9; *p* = 0.320; I^2^ = 97.7%, *p* < 0.001) and considering pre- vs. post-supplementation levels (MD: 7.1 mg/dL (2.5 µmol/L); 95% CI: −0.2, 14.4; *p* = 0.055; I^2^ = 0%, *p* = 0.361).

A significant reduction in apo B levels was found after administration of grape products as compared with placebo (MD: −2.4 mg/dL (−0.05 µmol/L); 95% CI: −4.5, −0.3; *p* = 0.026) with a non-significant heterogeneity among studies (I^2^ = 62.8, *p* = 0,068). However, in the intervention group, no significant change in apo B was found after supplementation with grape products as compared with pre-supplementation levels (MD: −3.2 mg/dL (−0.06 µmol/L); 95% CI: −8.2, 1.9; *p* = 0.218) without heterogeneity among studies (I^2^ = 0%, *p* = 0.993).

### 3.5. Publication Bias

Visual inspection of funnel plots and the Egger’s test suggested the absence of publication bias and of small-study effect for studies evaluating TC, HDL-C, and LDL-C (Egger’s *p* = 0.592, 0.906, and 0.761, respectively; Figure S5). In contrast, a significant publication bias was found for studies evaluating TG (Egger’s *p* = 0,018, Figure S6). The Duval and Tweedie’s trim and fill analysis (Supplemental Figure S6) showed that after adjusting for publication bias an even higher difference in TG was confirmed in patients receiving grape supplementation as compared to placebo (MD: −15.7 mg/dL (−0.18 mmol/L), 95% CI: −19.0, −12.4).

## 4. Discussion

The results of the present meta-analysis show that the administration of grape products is associated with a significant improvement of lipid profile, as evidenced by changes in TC, HDL-C, LDL-C, oxLDL-C, apo B, and TG levels in subjects receiving grape products compared to placebo. With regard to the extent of the lipid-lowering effect, the reduction was −5.6 mg/dL (−0.14 mmol/L) for LDL-C and −5.0 U/L for oxLDL-C. Although the magnitude of the effect is not impressive in absolute terms, it may still be noteworthy for the prevention of CV diseases on a population basis. Indeed, for each 1% decrease in LDL-C, there is a 1% decrease in cardiovascular event rate [62]. Of particular clinical relevance is also the reduction in oxLDL-C—an important player in the atherosclerotic process.

To the best of our knowledge, this is to date the largest meta-analysis evaluating the relationship between grape product intake and lipid profile. In a recent systematic review, Woerdeman et al. concluded that grape polyphenols seemed not to induce any relevant benefit on glycemia, blood pressure, and lipid levels in individuals with or without characteristics of the metabolic syndrome [63]. Differently, in the present study we found a favorable effect of grape supplementation on lipid profile. In addition, the meta-regression analysis showed that the presence of metabolic diseases, such as diabetes, obesity, and dyslipidemia, did not affect the differences in the changes of the lipid fractions, suggesting that the benefits of grape supplementation take place in individuals with or without metabolic diseases.

A critical factor to be considered in examining the currently available literature on grape polyphenols and cardiovascular benefits relates to the dose of grape product supplementations. In fact, the dose is quite variable among the studies analyzed, ranging from 22.4 to 2370 mg/day; thus, it cannot be excluded that in some studies, the dose of grape supplementation was too low to exert measurable effects, which could erroneously lead to the conclusion of negative results. The importance of the dose clearly emerges from our study; in fact, the subgroup analyses show that the reduction in LDL-C reached statistical significance when the daily grape polyphenols supplementation was >400 mg/day. This finding should be taken into account in supporting clinical recommendations as well as in designing future intervention studies.

The biological mechanisms underlying the favorable impact of grape products on lipid levels remain largely speculative. It is widely recognized that polyphenolic compound can inhibit pancreatic lipase, resulting in reduced fat digestion and absorption, with consequent reduced secretion of triglycerides rich lipoproteins in the hepatocytes [64]. In addition, grape polyphenols could reduce lipoprotein synthesis in hepatocytes by decreasing acyl-Coenzyme A cholesterol acyltransferase, inhibiting microsomal triglyceride transfer protein (MTP), and increasing fatty acid oxidation [65]. In addition, polyphenols could influence LDL-C composition as well as the cholesterol/triglyceride ratio by inhibiting the cholesterol ester transfer protein (CETP) [66]. With regard to the reduction in oxLDL-C, there is evidence that polyphenols are able to prevent LDL oxidation through their radical-trapping effects and their function as hydrogen donors to α-tocopherol radicals [67]. Interestingly, Toaldo et al. have recently shown in healthy subjects that the acute consumption of grape juice promoted a significant decrease in thiobarbituric acid reactive substances (TBARS)—an index of lipid peroxidation levels—when compared with the control intervention [68]. In line with this finding, Sano et al. demonstrated a significant reduction in the concentration of oxLDL-C, measured as plasma malondialdehyde after a 12-week administration of grape seed extracts in healthy subjects [46].

This meta-analysis enabled us to evaluate the individual impact of various grape products on lipid profile. As known, phenolic compounds are mainly distributed in the skin, stem, leaf, and seed of the grape, with juice having a lower phenolic content. Furthermore, the types of phenolic compounds vary with cultivar, soil composition, geographic origin, and also in relation to the different parts of the grape; i.e., seeds are rich in gallic acid, (+)-catechin, epicatechin, dimeric procyanidin, proanthocyanidins, while the skin is rich in proanthocyanidins, ellagic acid, myricetin, quercetin, kaempferol, trans-resveratrol [69]. In our study, we found a favorable effect of whole grape products on lipid profile, while no significant change was observed after supplementation with grape seed extracts and grape juice. This finding could be explained by the fact that whole grape products include skin, pomace, and seeds, which supply a mixture of polyphenol compounds with a potential synergistic effect on lipid metabolism [69]. From a clinical point of view, the results of this study support the nutraceutical-based approach as a useful complement to nutritional and pharmacological therapies to improve lipid profile, as stated in recently published guidelines [70,71]. With regard to the lipid-modifying effects of grape products, the present data indicate that a consumption of polyphenols derived from whole grape (i.e., pomace) at a minimum dose of 400 mg/day is required to have a significant reduction in LDL-C and oxLDL-C.

Some potential limitations of our study need to be discussed. First, the studies included in our meta-analysis have different inclusion and exclusion criteria and most subjects had concomitant CV risk factors. Therefore, we performed meta-regression analyses in order to adjust the results by demographic and clinical variables. In addition, since the meta-analysis was performed on aggregate data and there could be some missing information in each study, the multivariate approach allowed for the adjustment of some, but not all, potential confounders. Thus, we cannot exclude the influence of other factors on the outcomes of interest. Furthermore, the studies analyzed presented some variability in (1) type of supplementation, (2) polyphenol content, (3) study duration, and (4) study design. To limit these sources of variability, we performed subgroup analyses specifically stratifying for type of grape supplementation and polyphenols content. Similarly, we introduced study duration among the meta-regression variables, showing that this parameter had no impact on the results. With regard to the study design, although we included only RCTs, a potential source of bias was represented by the type of placebo used in the various studies since in some of them the control group received a placebo while in others no dummy supplementation was administered. Lastly, no data on the effect of grape supplementation on clinical outcomes are available in the studies included. Several studies indicate that the dietary intake of grape polyphenols is associated with an improvement of endothelial function, suggesting a cardioprotective effect of these compounds [24,72]. However, specific studies evaluating cardiovascular and cerebrovascular outcomes are needed to address this issue.

In conclusion, the present meta-analysis shows that grape polyphenols exert a favorable effect on lipid profile in humans by significantly reducing plasma levels of LDL-C and oxLDL-C. The effect is observed with a daily grape polyphenol supplementation >400 mg/day provided by whole grape products, but not grape seed extracts or grape juice. The lipid-lowering effect of grape polyphenols seems to occur in healthy subjects as well as in subjects with a range of metabolic abnormalities. Additional trials specifically in patients with dyslipidemia or diabetes mellitus are required to confirm this finding.

## Figures and Tables

**Table 1:** Characteristics of included studies.

Author	Study Design	Population (*n*)	Follow-up (weeks)	Type of Grape Product	Type of Control	Reported Outcomes	Age (years)	Male Gender (%)
Argani 2016 [47]	RCT-parallel double-blind	70 mild to moderate hyperlipidemia	8	SE	placebo	TC, HDL-C, TG, LDL-C, apo A	47.0	32.7
Banini 2006 [48]	RCT-parallel open	23 T2DM	4	J	no supplement	TC, HDL-C, TG, LDL-C	53.9	47.6
Dohadwala 2010 [49]	RCT-crossover double-blind	64 preHT/stage 1 HT	8	J	placebo	TC, HDL-C, TG, LDL-C	42.6	68.8
Han 2016 [50]	RCT-parallel double-blind	50 healthy subjects	10	WG	placebo	apo B	NA	47.9
Hansen 2005 [51]	RCT-parallel double-blind	35 healthy subjects	4	WG	placebo	TC, HDL-C, TG, LDL-C	52.0	45.6
Hollis 2009 [52]	RCT-parallel open	50 healthy subjects	12	J	no supplement	TC, HDL-C, TG, LDL-C	25.0	NA
Jiménez 2008 [53]	RCT-parallel open	43 non-smokers	16	WG	no supplement	TC, HDL-C, TG, LDL-C	35.3	37.2
Kar 2009 [29]	RCT-crossover double-blind	32 T2DM	4	SE	placebo	TC, HDL-C, TG	62.0	50.0
Martínez-Maqueda 2018 [33]	RCT-crossover open	98 subjects with MetS **	6	WG	no supplement	TC, HDL-C, TG, LDL-C	42.6	55.1
Mellen 2010 [54]	RCT-crossover double-blind	50 subjects with CAD or ≥1 CV risk factor	4	SE	placebo	TC, HDL-C, TG, LDL-C	52.1	50.0
Millar 2018 [55]	RCT-crossover double-blind	40 subjects with MetS	4	WG	placebo	TC, HDL-C, TG	53.5	60
Park 2009 [56]	RCT-parallel double-blind	40 healthy subjects	8	J	placebo	TC, HDL-C, TG, LDL-C	44.4	100.0
Preuss 2000 [35]	RCT-parallel double-blind	19 subjects with hyperlipidemia	8	SE	placebo	TC, HDL-C, LDL-C	NA	NA
Sano 2007 [46]	RCT-parallel single-blind	35 subjects with hyperlipidemia	12	SE	placebo	TC, HDL-C, TG, LDL-C, apo A, apo B	53	47.5
Siasos 2013 [57]	RCT-crossover double-blind	26 healthy smokers	2	J	placebo	TC, LDL-C	26.3	38.5
Sivaprakasapillai 2009 [27]	RCT-parallel double-blind	18 subjects with MetS	4	SE	placebo	TC, HDL-C, TG, LDL-C, oxLDL-C	46.5	38.5
Taghizadeh 2016 [58]	RCT-parallel double-blind	40 healthy females	8	SE	placebo	TC, HDL-C, TG, LDL-C	20.7	0.0
Tomé-Carneiro 2012 [59]	RCT-parallel triple-blind	50 T2DM or hyperlipidemia under statins	24	WG	placebo	TC, HDL-C, TG, LDL-C, oxLDL-C, apo B	59.5	54.0
Urquiaga 2015 [32]	RCT-parallel open	38 male with MetS *	16	WG	no supplement	HDL-C, TG	44.0	100.0
Vaisman 2015 [45]	RCT-parallel double blind	32 heterogeneous ^§^	12	WG	placebo	TC, HDL-C, TG, LDL-C	57.0	74.4
Vigna 2003 [34]	RCT-crossover double-blind	24 healthy males heavy smokers	4	WG	placebo	TC, HDL-C, TG, LDL-C	54.0	100.0
Yubero 2013 [60]	RCT-parallel double-blind	60 healthy subjects	8	SK	placebo	TC, HDL-C, LDL-C, oxLDL-C	51.0	NA
Zern 2005 [44]	RCT-crossover single-blind	44 pre-/postmenopausal women	4	WG	placebo	TC, HDL-C, TG, LDL-C	39.7 (premenopausal) 58.5 (postmenopausal)	0.0
Zunino 2014 [61]	RCT-crossover double-blind	24 obese	3	WG	placebo	HDL-C, TG, LDL-C, oxLDL-C	36.0	33.3

RCT: randomized controlled trial; SE: grape seed extract; J: grape juice; WG: whole grape product; SK: grape skin extract; TC: total cholesterol; HDL-C: high-density lipoprotein cholesterol; LDL-C: low-density lipoprotein cholesterol; TG: triglycerides; oxLDL-C: oxidized low-density lipoprotein cholesterol; apo A: apolipoprotein A; apo B: apolipoprotein B; NA: not available; T2DM: type 2 diabetes mellitus; HT: hypertension; MetS: metabolic syndrome. * At least one MetS criteria; ** at least two MetS criteria; ^§^ 35–70 years, BMI < 40 kg/m^2^, systolic blood pressure <154 mmHg and diastolic blood pressure <93 mmHg.

## Supplementary Materials

The following are available online at https://www.mdpi.com/2077-0383/9/2/313/s1, Figure S1: PRISMA Flow Diagram, Figure S2: Changes in TC levels after administration of grape products as compared with controls, Figure S3: Changes in HDL-C levels after administration of grape products as compared with controls, Figure S4: Changes in TG levels after administration of grape products as compared with control, Figure S5: Funnel plots of effect size versus standard error for studies evaluating levels of TC (panel A), HDL-C (panel B), LDL-C (panel C) levels after administration of grape products or placebo, Figure S6: Funnel plots of effect size versus standard error (panel A) and Duval and Tweedie’s trim and fill analysis (panel B) for studies evaluating levels of TG after administration of grape products or placebo, Table S1: Meta-regression analyses for TC, HDL-C, TG and LDL-C.
