# Supplementary material for: Impact of Grape Products on Lipid Profile: A Meta-Analysis of Randomized Controlled Studies
Source: J Clin Med. 2020 Jan 22;9(2):313. doi: 10.3390/jcm9020313 (PMC7073656; doi:10.3390/jcm9020313)
Supplement: Supplementary file 1 [file jcm-09-00313-s001.pdf]

Supplemental material

**Figure S1.**	PRISMA Flow Diagram
**Figure S2.**	Changes in TC levels after administration of grape products as compared with controls
**Figure S3.**	Changes in HDL-C levels after administration of grape products as compared with controls.
**Figure S4.**	Changes in TG levels after administration of grape products as compared with control
**Figure S5.**	Funnel plots of effect size versus standard error for studies evaluating levels of TC (panel A), HDL-C (panel B), LDL-C (panel C) levels after administration of grape products or placebo.
**Figure S6.**	Funnel plots of effect size versus standard error (panel A) and Duval and Tweedie's trim and fill analysis (panel B) for studies evaluating levels of TG after administration of grape products or placebo.
**Table S1.**	Meta-regression analyses for TC, HDL-C, TG and LDL-C.

Figure S1. PRISMA Flow Diagram

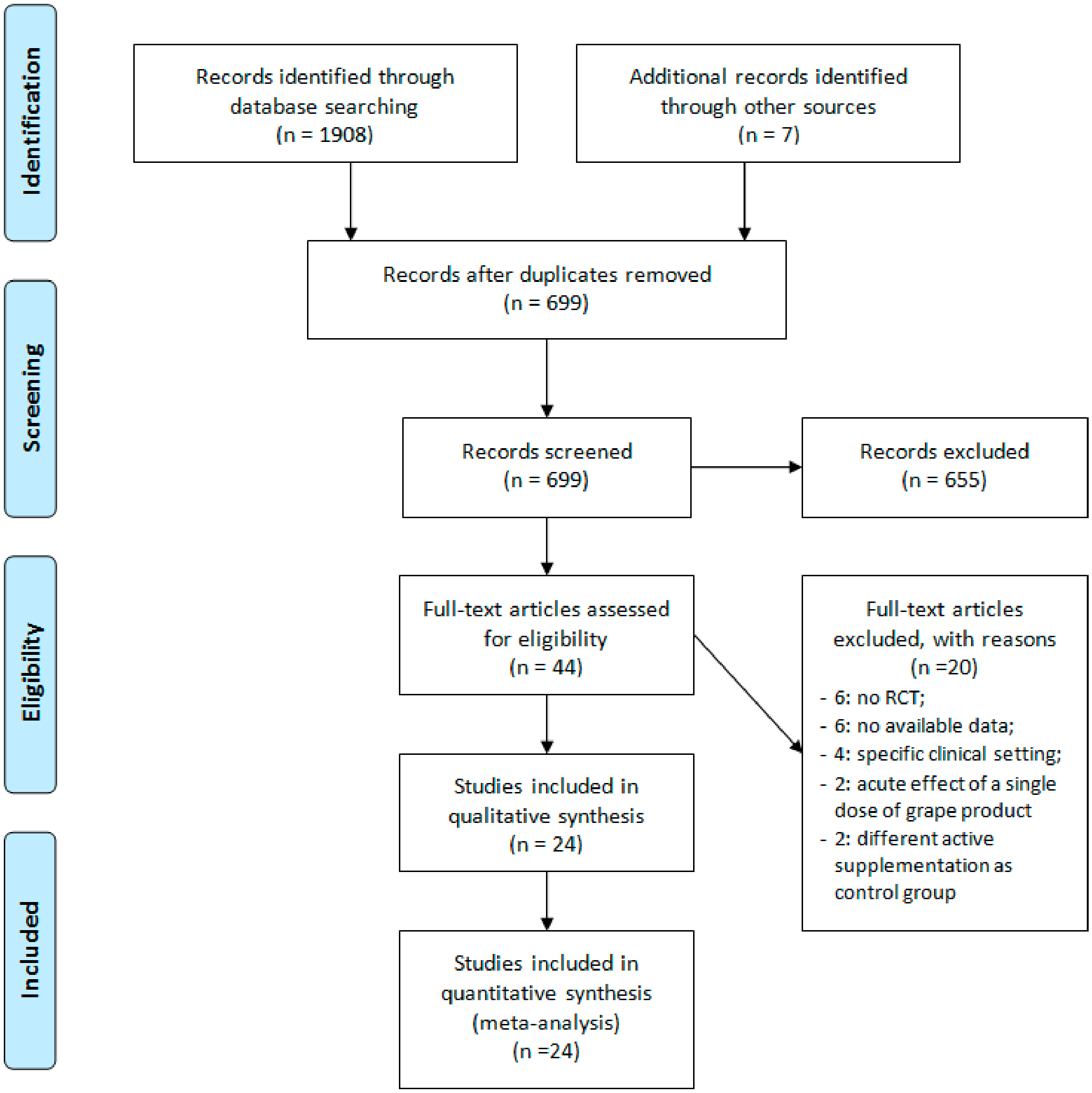

Figure S2. Changes in TC levels after administration of grape products as compared with controls

Study name	Statistics for each study: Difference in means	Lower limit	Upper limit	p-Value
Argani 2016	-25,000	-28,462	-21,538	0,000
Banini 2006	-4,800	-10,428	0,828	0,095
Dohadwala 2010	-2,000	-4,533	0,533	0,122
Hansen 2005	-8,500	-28,244	11,244	0,399
Hollis 2009	-15,000	-17,531	-12,469	0,000
Jiménez 2008	-19,100	-28,146	-10,054	0,000
Kar 2009	-4,000	-6,567	-1,433	0,002
Martinez-Maqueda 2018	-4,000	-5,731	-2,269	0,000
Mellen 2010	-1,300	-3,650	1,050	0,278
Millar 2018	-11,000	-16,205	-5,795	0,000
Park 2009	-1,300	-8,037	5,437	0,705
Preuss 2000	-3,000	-12,511	6,511	0,536
Sano 2007	0,000	-5,235	5,235	1,000
Siasos 2013	-4,000	-12,188	4,188	0,338
Sivaprakasapillai 2009	-4,000	-6,741	-1,259	0,004
Taghizadeh 2016	-4,000	-7,434	-0,566	0,022
Tomé-Carneiro 2012	-10,900	-16,180	-5,620	0,000
Vaisman 2015	-4,500	-7,736	-1,264	0,006
Vigna 2003	-8,000	-11,038	-4,962	0,000
Yubero 2013	-25,800	-28,954	-22,646	0,000
Zern 2005 (a)	-4,000	-7,405	-0,595	0,021
Zern 2005 (b)	-4,000	-8,493	0,493	0,081
	-7,625	-10,832	-4,419	0,000

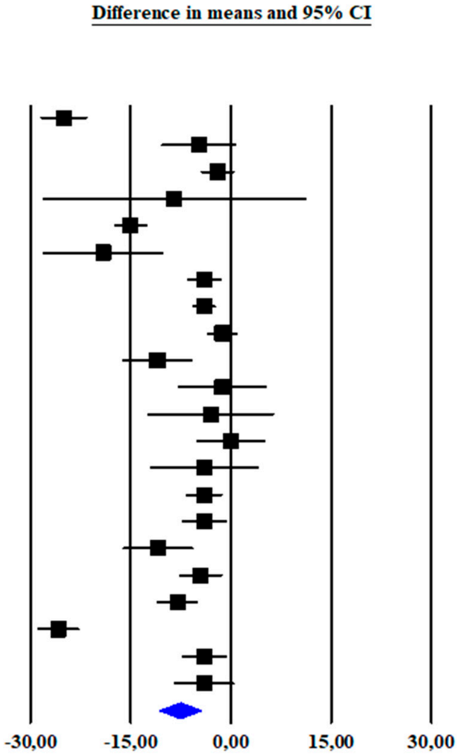

Figure S3. Changes in HDL-C levels after administration of grape products as compared with controls.

Study name	Difference in means	Lower limit	Upper limit	p-Value
Argani 2016	7,300	-12,995	27,595	0,481
Banini 2006	0,700	-1,975	3,375	0,608
Dohadwala 2010	1,000	-0,326	2,326	0,140
Hansen 2005	1,500	-5,929	8,929	0,692
Hollis 2009	2,000	0,961	3,039	0,000
Jiménez 2008	2,500	0,786	4,214	0,004
Kar 2009	0,000	-0,854	0,854	1,000
Martinez-Maqueda 2018	2,000	-1,315	5,315	0,237
Mellen 2010	0,700	0,126	1,274	0,017
Millar 2018	0,900	-1,264	3,064	0,415
Park 2009	2,000	1,271	2,729	0,000
Preuss 2000	1,600	-1,062	4,262	0,239
Sano 2007	0,200	-1,461	1,861	0,813
Sivaprakasapillai 2009	3,000	2,255	3,745	0,000
Taghizadeh 2016	4,000	2,218	5,782	0,000
Tomé-Carneiro 2012	1,500	0,415	2,585	0,007
Urquiaga 2015	1,900	-0,578	4,378	0,133
Vaisman 2015	0,900	-0,907	2,707	0,329
Vigna 2003	0,100	-0,848	1,048	0,836
Yubero 2013	2,500	1,870	3,130	0,000
Zern 2005 (a)	0,000	-0,805	0,805	1,000
Zern 2005 (b)	0,000	-1,260	1,260	1,000
Zunino 2014	2,170	0,224	4,116	0,029
	1,371	0,840	1,902	0,000

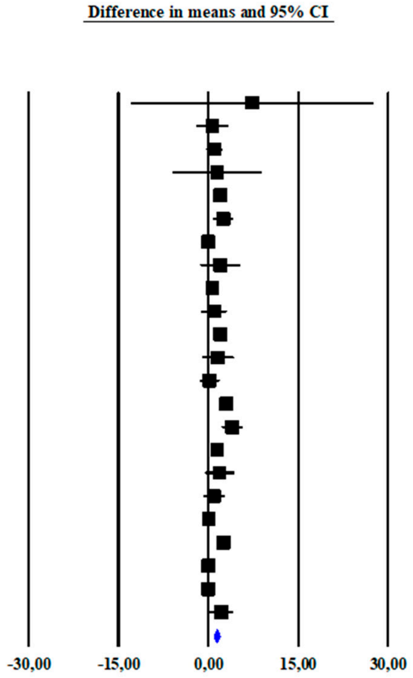

Figure S4. Changes in TG levels after administration of grape products as compared with controls

Study name	Difference in means	Lower limit	Upper limit	p-Value
Argani 2016	-30,700	-34,957	-26,443	0,000
Banini 2006	-6,000	-16,575	4,575	0,266
Dohadwala 2010	-7,000	-15,522	1,522	0,107
Hansen 2005	-2,600	-19,094	13,894	0,757
Hollis 2009	-6,000	-21,494	9,494	0,448
Jiménez 2008	-19,900	-34,253	-5,547	0,007
Kar 2009	-26,900	-34,174	-19,626	0,000
Martínez-Maqueda 2018	-9,000	-10,195	-7,805	0,000
Mellen 2010	-6,800	-7,227	-6,373	0,000
Millar 2018	-49,000	-58,481	-39,519	0,000
Park 2009	-1,700	-39,292	35,892	0,929
Sano 2007	-13,000	-20,207	-5,793	0,000
Sivaprakasapillai 2009	-6,000	-9,356	-2,644	0,000
Taghizadeh 2016	-18,000	-20,367	-15,633	0,000
Tomé-Carneiro 2012	-11,800	-20,433	-3,167	0,007
Urquiaga 2015	-27,400	-39,451	-15,349	0,000
Vaisman 2015	-6,200	-14,294	1,894	0,133
Vigna 2003	-18,000	-29,297	-6,703	0,002
Zern 2005 (a)	-17,700	-24,131	-11,269	0,000
Zern 2005 (b)	-8,000	-18,961	2,961	0,153
Zunino 2014	-0,600	-8,035	6,835	0,874
	-14,475	-17,778	-11,172	0,000

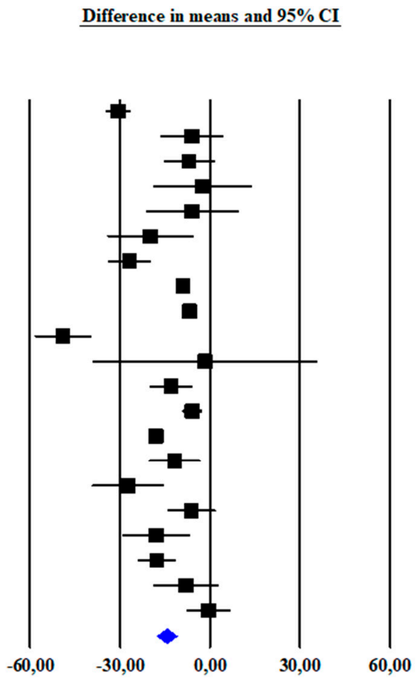

Figure S5. Funnel plots of effect size versus standard error for studies evaluating levels of TC (panel A), HDL-C (panel B), LDL-C (panel C) after administration of grape products or placebo.

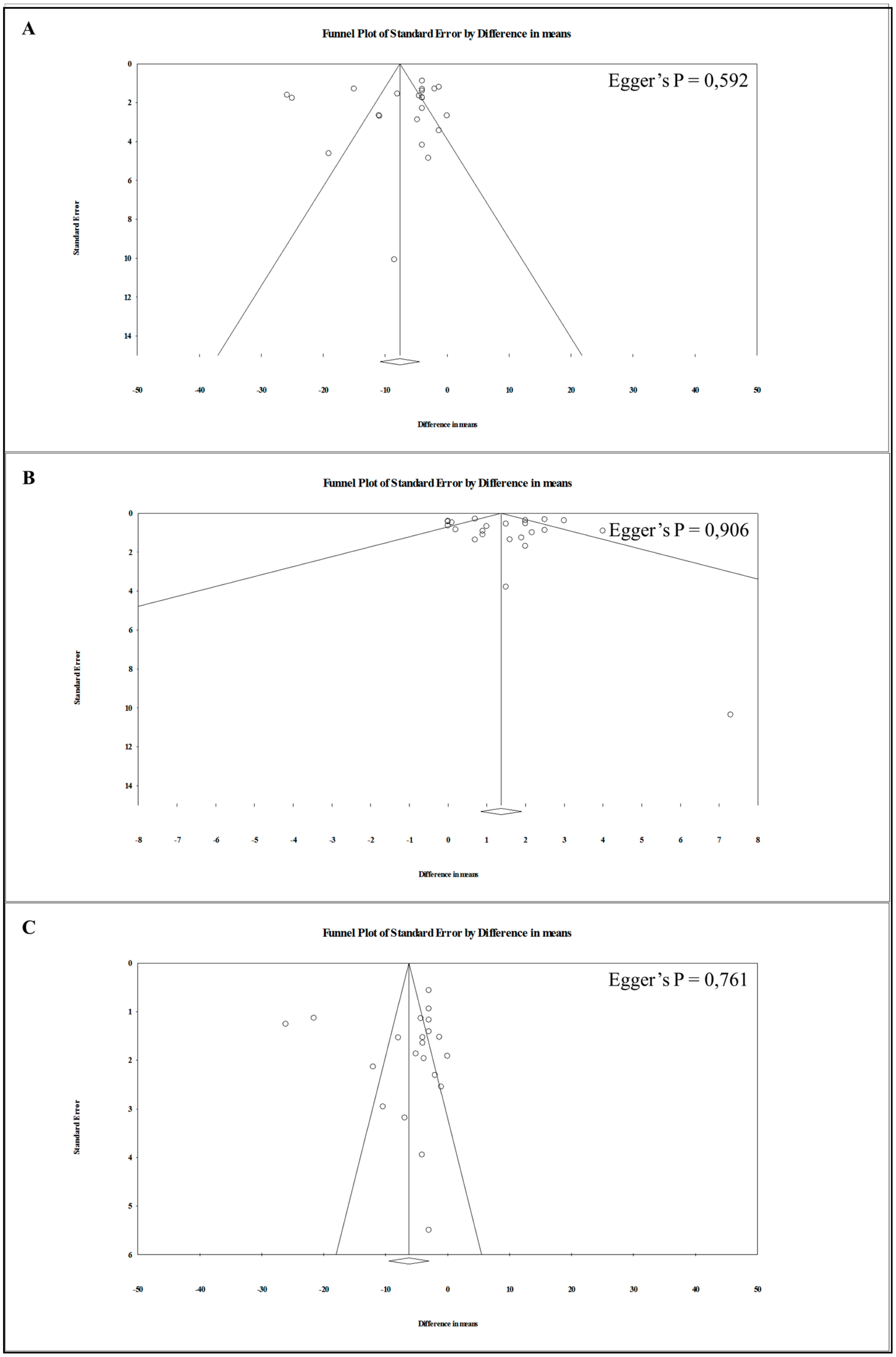

Figure S6. Funnel plots of effect size versus standard error (panel A) and Duval and Tweedie's trim and fill analysis (panel B) for studies evaluating levels of TG after administration of grape products or placebo.

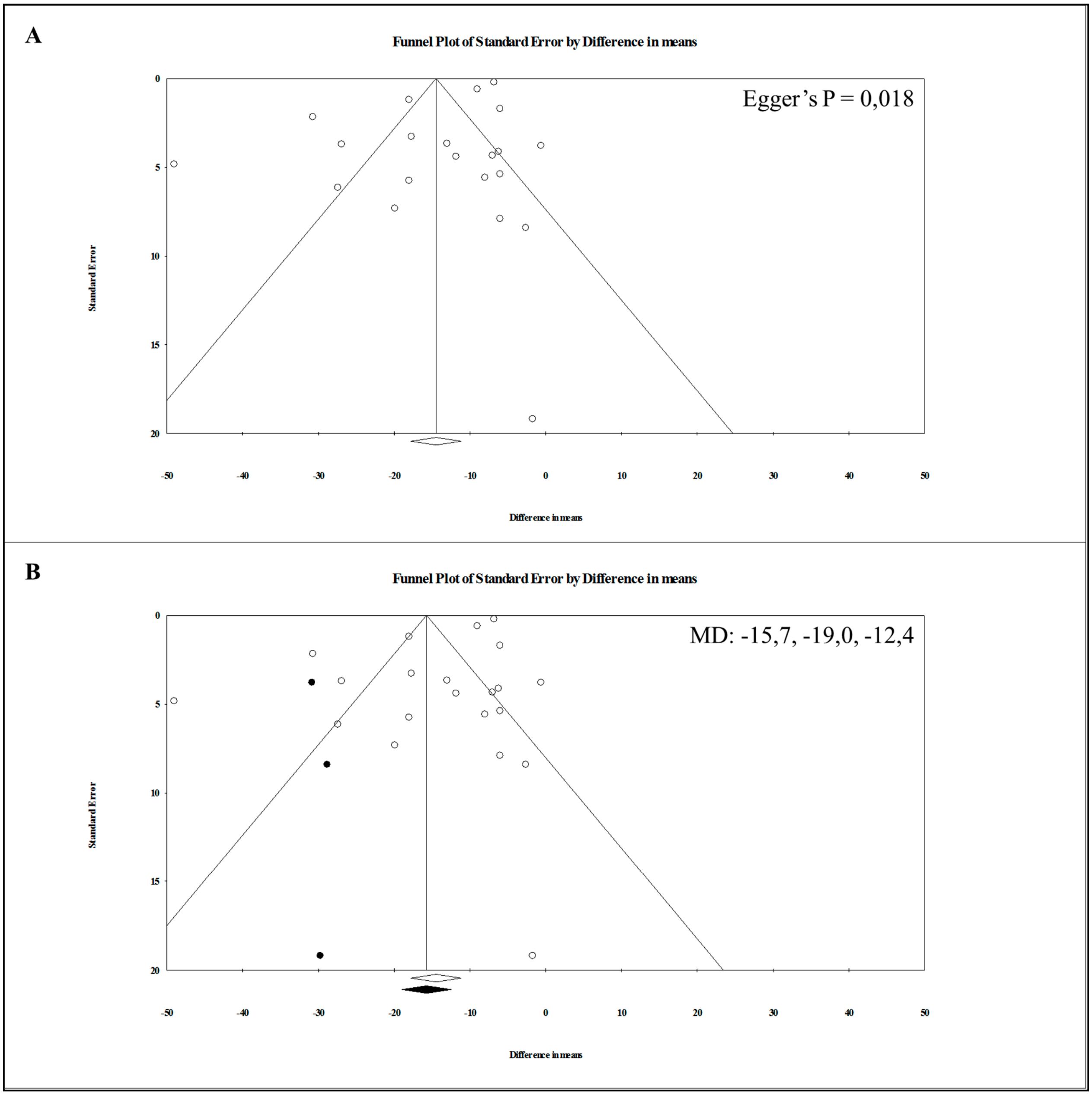

Table S1. Meta-regression analyses for TC, HDL-C, TG and LDL-C.

A) TC (22 datasets -21 studies)	Z-value	P-value
Age	0.29	0.77
Male gender	0.34	0.73
BMI	0.34	0.74
Diabetes	0.63	0.53
Dyslipidemia	0.19	0.85
Follow up	-1.33	0.19

B) HDL-C (23 datasets – 22 studies)	Z-value	P-value
Age	-2.58	**0.01**
Male gender	-0.19	0.85
BMI	-0.34	0.74
Diabetes	-1.54	0.12
Dyslipidemia	-0.51	0.61
Follow up	1.02	0.31

C) TG (21 datasets – 20 studies)	Z-value	P-value
Age	-0.59	0.56
Male gender	-0.34	0.73
BMI	0.82	0.41
Diabetes	-1.25	0.21
Dyslipidemia	-1.86	0.06
Follow up	-0,29	0.77

D) LDL-C (21 datasets – 20 studies)	Z-value	P-value
Age	-0.19	0.84
Male gender	0.97	0.33
BMI	-0.24	0.81
Diabetes	0.72	0.47
Dyslipidemia	-0.13	0.90
Follow up	0.23	0.82
